# ReformAlign: improved multiple sequence alignments using a profile-based meta-alignment approach

**DOI:** 10.1186/1471-2105-15-265

**Published:** 2014-08-07

**Authors:** Dimitrios P Lyras, Dirk Metzler

**Affiliations:** Faculty of Biology, Department II, Ludwig-Maximilians Universität München, Planegg-Martinsried, 82152 Germany

**Keywords:** Multiple sequence alignment, Iterative refinement, Dynamic programming

## Abstract

**Background:**

Obtaining an accurate sequence alignment is fundamental for consistently analyzing biological data. Although this problem may be efficiently solved when only two sequences are considered, the exact inference of the optimal alignment easily gets computationally intractable for the multiple sequence alignment case. To cope with the high computational expenses, approximate heuristic methods have been proposed that address the problem indirectly by progressively aligning the sequences in pairs according to their relatedness. These methods however are not flexible to change the alignment of an already aligned group of sequences in the view of new data, resulting thus in compromises on the quality of the deriving alignment. In this paper we present ReformAlign, a novel meta-alignment approach that may significantly improve on the quality of the deriving alignments from popular aligners. We call ReformAlign a meta-aligner as it requires an initial alignment, for which a variety of alignment programs can be used. The main idea behind ReformAlign is quite straightforward: at first, an existing alignment is used to construct a standard profile which summarizes the initial alignment and then all sequences are individually re-aligned against the formed profile. From each sequence-profile comparison, the alignment of each sequence against the profile is recorded and the final alignment is indirectly inferred by merging all the individual sub-alignments into a unified set. The employment of ReformAlign may often result in alignments which are significantly more accurate than the starting alignments.

**Results:**

We evaluated the effect of ReformAlign on the generated alignments from ten leading alignment methods using real data of variable size and sequence identity. The experimental results suggest that the proposed meta-aligner approach may often lead to statistically significant more accurate alignments. Furthermore, we show that ReformAlign results in more substantial improvement in cases where the starting alignment is of relatively inferior quality or when the input sequences are harder to align.

**Conclusions:**

The proposed profile-based meta-alignment approach seems to be a promising and computationally efficient method that can be combined with practically all popular alignment methods and may lead to significant improvements in the generated alignments.

**Electronic supplementary material:**

The online version of this article (doi:10.1186/1471-2105-15-265) contains supplementary material, which is available to authorized users.

## Background

The alignment of multiple DNA, RNA or Protein sequences is of major importance for a variety of biological modelling methods, including the estimation of the phylogenetic tree of the sequences and the prediction of their structural, functional and/or evolutionary relationships [1]. In addition, the recent advances in rapid, low-cost sequencing methods, have resulted in the accumulation of large amounts of molecular data to be processed, making thus the need for fast and accurate multiple sequence aligners even more imperative [2].

A widely used approach to cope with the Multiple Sequence Alignment (MSA) problem, is the employment of a computational formulation comprised of two major components, namely an objective function [3] able to quantify the degree of similarity of a given alignment and an optimization procedure that targets at identifying the optimal alignment based on the underlying objective function [4]. Concerning the former component, the Sum-of-Pairs scoring model (SP) [4, 5] remains amongst the most popular choices [6–8].

The maximization of the SP score is usually performed using dynamic programming. For the pairwise alignment case, an optimal (numerically but not necessarily biologically) solution can be found within reasonable time. However this does not hold for the multiple sequence alignment case, where it has already been shown [9–12] that obtaining the optimal alignment using the SP score is NP-hard. To overcome the computational intractability of the MSA problem, a large number of efficient heuristic algorithms have been proposed with the most popular being the progressive alignment approach [13–16].

In progressive alignment the sequences are initially placed on a bifurcating tree according to their degree of similarity. Then, they are progressively aligned in pairs following the formed guide tree in a bottom-up order until its root is reached. At each step, two nodes of the tree (i.e. two sequences, a sequence and an alignment or two alignments) are aligned by a standard pairwise alignment algorithm, and the deriving subalignment is retained to be used at a subsequent step. One important aspect of the progressive alignment strategy is the “once a gap, always a gap” rule, first introduced in [13]. Based on this policy, once a group of sequences is aligned, all gaps in the alignment are replaced by a neutral ‘X’ symbol ensuring that all subsequent pairwise alignments will be consistent with the pre-existing alignment of the group [17]. This rule by definition implies that once a group of alignments has been built up, they will remain fixed even in the view of new sequences that could potentially improve the overall alignment. Consequently, early errors in the progressive alignment steps are accumulated and propagated to later alignment stages compromising thus the alignment quality.

This problem is often tackled by using iterative refinement techniques [18, 19]. In iterative refinement, one sequence (or a group of sequences) is iteratively subtracted and realigned against the alignment of the remaining sequences. Via this sequence-profile or profile-profile realignment, a new alignment is obtained which is then used for the next iteration of the algorithm. The refinement terminates when a fixed number of iterations is reached or when the alignment remains unchanged between consecutive iterations [17]. Although these methods are very efficacious at curating early alignment errors, they only partially address the “frozen subalignments” issue, since at each iteration of the algorithm only one sequence (or a group of sequences) is realigned whereas the alignment of the remaining sequences is kept fixed.

In this article we present a variation of the aforementioned iterative refinement strategy where all sequences may be simultaneously and independently realigned against a summarization profile that encapsulates all the starting alignment information. The process begins by constructing a standard profile that summarizes all the initial alignment information. Then, a series of individual (and possibly concurrent) sequence-profile pairwise comparisons takes place, recording the way that each sequence is aligned against the profile. The new alignment is then *indirectly inferred* by merging all the individual subalignments into a unified group. The proposed approach is implemented as part of a newly introduced meta-aligner under the name ReformAlign (**Reform**ed **Align**ments) and is freely available to the public from http://evol.bio.lmu.de/_statgen/software/reformalign/ under the GNU General Public License (version 3 or later). We call ReformAlign a meta-aligner, in the sense described in [20], meaning that an initial alignment is required and it can be used with a variety of alignment programs.

As currently ReformAlign can only align DNA/RNA sequences, for the needs of our performance evaluations real nucleic acid datasets of variable length and average sequence identity rates were used. Our experimental results demonstrate that the suggested profile-based modification of the classic progressive alignment and iterative refinement strategies is able to overcome the challenges posed by the propagation of early pairwise alignment errors and that ReformAlign is an efficient, well suited approach that may improve on the performance of a vast variety of existing alignment software.

## Implementation

### Alignment strategy

ReformAlign aims at improving on the quality of an existing alignment by providing the sequences with an additional opportunity to be individually re-aligned against a standard profile that efficiently summarizes the starting alignment information. The idea is that via this re-alignment step, early alignment errors caused by “frozen” subalignments may be fixed, delivering thus better results in terms of alignment accuracy.

Towards this end, in ReformAlign the alignment of the sequences is performed in two steps. The first step involves the construction of a non-probabilistic profile from an existing alignment, whereas during the second step all the sequences are individually realigned against the profile that derived from the first step. The new alignment is finally indirectly reconstructed by merging all the individual sequence-profile subalignments into a unified group. Due to the nature of the latter step, all pairwise sequence-profile comparison can be performed in parallel, improving thus the aligner performance in terms of execution time.

**Figure 1:**
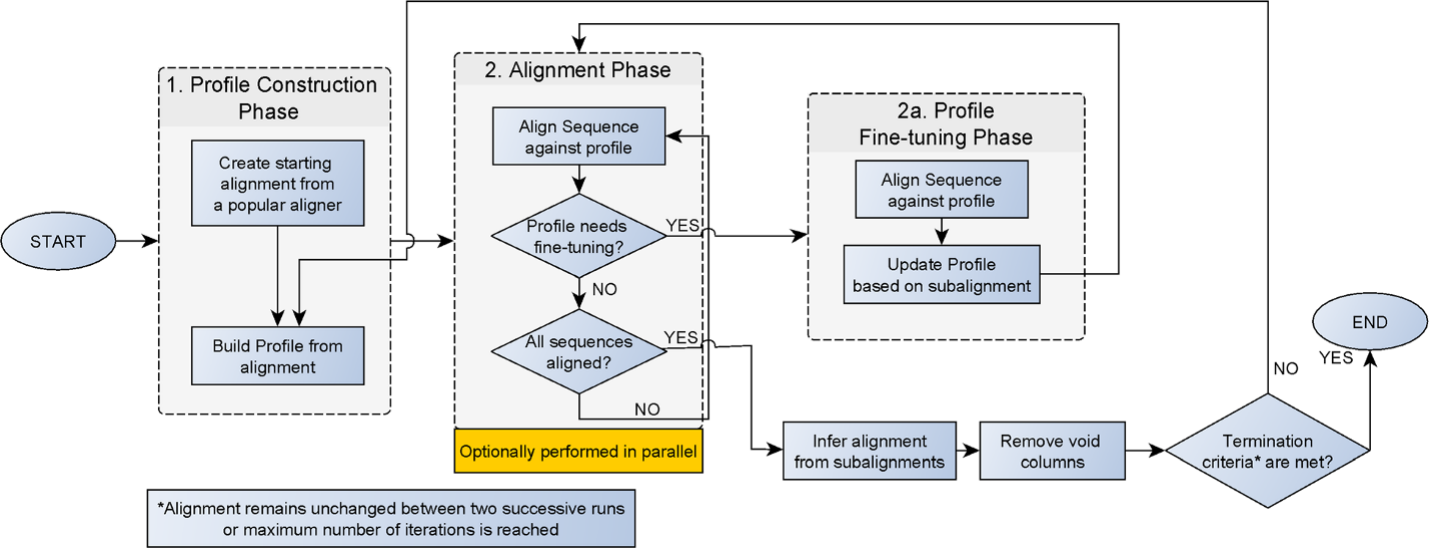
**Logic Flow diagram of the ReformAlign alignment strategy.** The algorithm creates an initial profile based on a starting alignment deriving from an established aligner. Then, all the sequences are aligned against the formed profile to obtain the reformed alignment. Notice that in this step, in case a new insertion is detected, the algorithm automatically switches to a profile fine-tuning mode in order to modify the profile to also account for the new insertion(s). After all sequences have been successfully aligned against the profile, the deriving alignment is indirectly inferred by merging all the estimated subalignments into a unified set and it is then freed from uninformative void columns (i.e. columns consisting exclusively of gaps). The process is repeated using the reformed alignment as starting alignment until the alignment between two successive runs remains unchanged or a predefined maximum number of iterations is reached.

Upon completion of the latter step, a new (reformed) alignment is derived which is very often different to the starting one. Since this alignment may be also susceptible to further improvement, the whole process can be re-initiated using the reformed alignment as starting alignment. The algorithm terminates when either the alignment between two successive runs remains unchanged or a pre-defined maximum number of iterations is reached. A diagrammatic overview of the ReformAlign alignment logic flow is provided at Figure 1.

#### A. Profile construction step

In the profile construction step, a popular aligner is employed to infer a starting alignment for the examined group of sequences. Then, a summarization profile is constructed upon the starting alignment, having as many position as the columns in the alignment. Each position in the profile is characterized by a series of < Residue, Weight > pairs sized to fit the number of distinct residues that appear in the corresponding column of the alignment. The “Residue” part in each pair corresponds to the label of a particular residue in the alignment column whereas its “Weight” represents the number of times that this particular residue appears in that column.

#### B. Sequence alignment step

In ReformAlign, the formation of the final alignment is performed indirectly by aligning all the sequences against the profile that derived from the first step. All sequence-profile alignments are performed using a standard pairwise alignment algorithm and for each sequence its alignment against the profile is recorded. By joining all deriving pairwise alignments into a unified group, it is possible to infer the final alignment of the examined sequences. In contrast to traditional iterative refinement approaches where at each refinement only one sequence (or a group of sequences) is realigned, in the proposed approach all the sequences have a chance to align differently against the formed summarization profile.

Intuitively, the majority of the pairwise alignments that will come up from this step will have at most as many columns as the number of positions of the formed profile. Nevertheless this is not always the case. For example, it might happen that a particular residue is aligned as a mismatch in one of the columns of the starting alignment, but during the sequence alignment step the score obtained by misaligning this residue against the corresponding profile position might be smaller than the penalty for opening a new gap in the alignment. In cases where the profile has to be updated in order to accommodate new insertion(s), ReformAlign automatically switches to a profile fine-tuning mode. As soon as the profile has been successfully updated, ReformAlign restarts the sequence alignment step using the new profile.

Another important aspect of the suggested meta-alignment technique is that since the alignment of the sequences may alter during the sequence alignment step, it might happen that none of the sequences aligns against one or more of the positions of the final profile, leading thus to the derivation of void (all gapped) columns. To fix for this issue, upon completion of the sequence alignment step, the program checks for uninformative void columns and completely removes them from the reported alignment.

#### C. Iterative refinement

After the sequence alignment step has been completed, a new alignment is produced. In case the reformed alignment is different to the starting one, ReformAlign may be iteratively applied to further fine-tune the produced alignment(s). The iterative refinement step terminates when the pre-defined limit of iterations is reached or when the deriving alignments between two successive runs of the algorithm are identical.

### Dynamic programming and scoring system

The dynamic programming scheme employed by ReformAlign for the sequence-profile alignment task is the Gotoh’s [21] affine-gap penalties variation of the Needleman-Wunsch global alignment algorithm [22], as described in [17].

Regarding the scoring scheme for assessing the similarity between a position in a sequence and one from the profile, in contrast to the average function encountered in various alignment models such as ClustalW [6], we employed an additive variant where the observed counts of the residues are used instead of their averaged frequencies, as follows:


where **A** is the current alphabet (e.g. **Α** = {*A*, *C*, *G*, *T*} for DNA alignments), *C*_*a*_ and *C*_*b*_ represent the observed counts (tallies) of residues *a* and *b* respectively and *Sub*_*a* → *b*_ corresponds to the score matrix value for aligning residue *a* to *b*.

The main reason for selecting aforementioned scoring scheme is that the use of the observed counts instead of the relative frequencies allows for a better discrimination between well-conserved columns. For example, a profile position exclusively comprised of a several Cytosine residues will have exactly the same score as another profile position formed by a single Cytosine, if the averaged sum of pairs scoring scheme was used, but the score for the former case would be significantly higher if the additive scoring model was employed instead.

### Alignment parameters

The choice of a proper values for the alignment parameters is essential for the multiple sequence alignment problem, since it has been proven [23, 24] that it may significantly affect the decision of the “optimal” alignment among the numerous alternatives. These parameters may significantly vary among different alignment approaches and they are usually decided by cross validation testing.

In ReformAlign, the alignment parameters that have to be defined are the substitution scores and the gap opening and extension penalties. However, it would be desirable in our strategy that each residue of the sequences could inexpensively skip any low-scoring profile positions in order to align itself against another higher scoring position. Furthermore, based on the fact that by definition the profile already incorporates enough positions where every residue of the sequences could possibly be aligned, opening a new gap in the profile should be more costly than opening a gap to the sequence. Consequently, in ReformAlign four distinct types of gap penalties are considered: a penalty for opening a gap in the profile (HGOP), a penalty for extending a gap in the profile (HGEP), a penalty for opening a gap in the sequence (VGOP) and a penalty for extending a gap in the sequence (VGEP). As in ClustalW, terminal gaps (TGOP and TGEP) are not penalized in our approach either.

An additional parameter constraint deriving from the inherent properties of our model is that the substitution scores should be substantially higher than the gap penalties. In case this restriction is not met, it might happen that many sequences require a profile update during the sequence alignment phase, resulting thus in very large profiles containing a lot of poorly supported positions. To avoid such cases it is recommended that substitution scores are always set to (relatively high) positive values whereas gap opening and extension penalties are set to negative values.

Regarding the substitution weights, ReformAlign currently uses a modified version of the HOXD substitution matrix [25] where all scores are increased by a positive “bonus” value, so that the aforementioned condition is met and to further reward (mis)matches compared to gaps. Nevertheless, increasing all substitution scores by the same “bonus” value results in understating the differences between matches and mismatches. For this reason, each score value is then increased by a re-adjustment coefficient (Coeff).

The default bonus value and re-adjustment coefficient applied to the score matrix as well as the default gap opening and extension penalties were decided based on a greedy parameters search that was performed using MUSCLE [8, 26] as the starting aligner and a separate training set with alignments from the data-set 1 of a previous version of BRAliBase (i.e. BRAliBase II) [27]. For each test in the training set, the percent APSI value of the initial alignment was calculated and then more than 300,000 parameter combinations were greedily assessed by comparing each time the deriving (reformed) alignment to the expected one (reference alignment) in terms of alignment accuracy. The results of this cross-validation analysis suggested that the score matrix bonus value and the gap penalties should be re-adjusted according to the APSI value of the initial alignment based on the subsequent formulas:


where *APSI*_*p*_ is the percent Average Pairwise Sequence Identity calculated over the initial alignment.

The aforementioned scheme for defining the starting parameter values was used for all the experiments outlined in the present study and is automatically employed by default in ReformAlign, unless more appropriate starting values are defined to the program by the user.

Finally, ReformAlign follows ClustalW’s paradigm [6] by heuristically updating the gap opening and extension penalties during runtime, in an attempt to deliver superior alignment results. In particular, the penalties are modified based on the following criteria:

#### Dependency on the lengths of the sequences

Both the gap opening and gap extension penalties are modified depending on the ratio between the length (number of residues) of the sequence to be aligned and the largest sequence in the set. Intuitively, gaps should be penalized harder for shorter sequences in order to avoid having alignments with too many and/or sparse gaps. The calculation of the starting gap penalties is performed according to the following formula:


where *GP* represents all types of gap penalties (HGOP, HGEP, VGOP and VGEP), *M* is the length of the largest sequence in the set and *N* is the length of the sequence to be aligned.

#### Position-specific penalties based on the profile conservation rate

During the computation of the dynamic programming matrix, the gap opening penalties (HGOP and VGOP) are modified in a position-specific way depending on the conservation rate of the profile. If a position in the profile is well conserved (the weights for one or more of its pairs are set to relatively high values), then this implies that the specific position is strongly supported by many sequences (probably even by the examined sequence itself – based on the starting alignment) and thus the penalty for opening a new gap against this profile position should be higher compared to weakly supported positions. Consequently, the gap opening penalties per position are calculated as follows:


where *GOP* represents the gap opening penalties (HGOP, and VGOP), *PCR*_*i*_ is the Profile Conservation Rate for the *i*^*th*^ position of the profile calculated as a fraction of the maximum weight value among all *< Residue, Weight >* pairs contained in the *i*^*th*^ position of the profile divided by the sum of all weights of the pairs in the *i*^*th*^ position and *SS*_*ij*_ is the substitution score for aligning the *i*^*th*^ position of the profile against the *j*^*th*^ position of the sequence.

The idea behind the multiplication of the profile conservation rate by the substitution score is to linearly increase the gap opening penalties for conserved columns, so that gaps would be favored only if subsequent matches of the sequence against ensuing positions of the profile would score high enough to sufficiently compensate for the very costly gap that was opened.

## Methods

In order to assess the efficiency of ReformAlign in improving the quality of existing alignments, testsets from the BRAliBase 2.1 RNA alignment database [27, 28] and the DNA SMART database [29] were used.

### BRAliBase 2.1

BRAliBase (Benchmark RNA Alignment database**)** is a collection of RNA alignments taken from the Rfam database. It was initially introduced in [30], but since then it has been further enriched with additional alignments [27, 28], leading to its current version v2.1 which contains in total 18,990 aligned sets of sequences (packed in sets of 2, 3, 5, 7, 10 and 15 sequences) with an Average Pairwise Sequence Identity (APSI) rate ranging between 20% and 95% [28]. For the needs of the present study we limited our analysis to the BRAliBase alignments composed of 7, 10 and 15 sequences per alignment.

### DNA SMART

The DNA Reference Alignment Benchmarks database [29] was proposed in 2007 as a collection of DNA reference alignments for the assessment of MSA applications. It is comprised of multiple DNA sequence alignments (MDSAs) corresponding to protein alignments of the BAliBASe [31], OXBench [32], PREFAB [8] and SMART [33] benchmarking datasets. For our experiments, we considered all alignments corresponding to the SMART database containing between 20 and 300 sequences per alignment. Since however ReformAlign does not currently support ambiguous characters, we limited our analysis to a total of 264 alignments containing exclusively unambiguous DNA letters.

### Accuracy measures

To assess the agreement between the reconstructed and the reference alignments, the following measures of accuracy were considered.

The sum of pairs (SP) score (aka developer’s score - *f*_*D*_ [34]) was initially proposed by Thompson *et al.* [35] and is defined as a fraction of the number of the correctly aligned residue pairs in the reconstructed alignment over the number of aligned residue-pairs of the reference alignment. If the denominator of the fraction is replaced by the number of residue-pairs in the test alignment, then the reverse sum of pairs score (aka modeler’s score - *f*_*M*_) is obtained. Finally, the total columns (TC) score is computed by dividing the number of correctly identified columns in the reconstructed alignment over the total number of columns in the reference alignment.

Although these metrics can provide an overview of the quality of the deriving alignments, each one has its own drawbacks. The developer’s score fails to penalize over-alignments and could give a great score to an alignment that erroneously aligns non-homologous regions, whereas the modeler’s score is not sensitive to detecting under-alignments and thus could possibly give high scores to alignments that systematically fail to align homologous regions (refer to [36] for further details regarding these types of alignment errors). Finally, the TC score is very sensitive to the misalignment of even a single sequence yielding a zero-valued TC Score even if all the remaining sequences in the examined group are properly aligned.

For this reason, two additional metrics were considered: a) the Cline’s score (CS) [36], which efficiently addresses the issues of the developer’s and modeler’s scores by penalizing over- and under- alignments while taking into account minor shifts in the reconstructed alignment compared to the reference and b) the D-POS metric proposed by Blackburne and Whelan [37] which satisfies the conditions of symmetry and triangular inequality that are not met by the SP and TC scores, where at the same time it incorporates information from indels by recording the position where gaps occur. All accuracy measures take their values in the [0,1] range, with the exception of Cline’s Score which may also take negative values in case there exist many large shifts. Moreover, in contrast to the remaining metrics, D-POS reports the distance between the generated and the reference alignment and thus lower D-POS values correspond to better alignments.

Since D-POS and Cline’s Score can efficiently summarize the quality of the deriving alignments while at the same time being the most immune to deficiencies, the discussion will be mostly based on the experimental results of these two metrics.

### Alignment programs

We assessed the effect ReformAlign on the deriving alignments of ten leading methods: ClustalW [38], ClustalO [39], MUSCLE [8, 26], MAFFT [40], Kalign [7], GramAlign [41], ProbConsRNA [42], R-Coffee [43, 44], PicXAA [45] and Dialign-TX [46]. These aligners implement a variety of alignment techniques, such as progressive alignment [7, 38, 41], iterative refinement [26, 39, 40], segment-based alignment [46], probabilistic/consistency-based alignment [42, 44] and maximum expected accuracy alignment [45]. Moreover, R-Coffee specializes in the alignment of RNA sequences and could thus provide us with an overview of the way that the proposed sequence-based method may affect initial alignments that use secondary structure information.

Since our main intention was to examine the effect of ReformAlign on existing alignments rather than to analytically assess the efficiency of each aligner on the benchmark databases, we used the default settings for all aligners except for MAFFT which was executed using both the –FFT-NS-i and the –L-INS-i options. Finally, the default value for the maximum number of refinement iterations in ReformAlign was set to 5 for all experiments (an analytical listing of the command-line options used for each aligner is provided at Additional file 1: Table S1).

## Results and discussion

Each benchmark test was given as input to all the considered aligners in order to obtain an initial alignment which was compared against the reference. Then, this alignment was provided as input to ReformAlign and the newly generated alignment was also compared against the reference using all considered accuracy metrics. Via this procedure, we came up with eleven distinct alignment pairs for each benchmark test (Mafft was separately assessed for the FFT-NS-i and L-INS-i options). Furthermore, in order to determine the statistical significance of the differences between the initial and the reformed alignments, Wilcoxon signed rank tests were also performed for each alignment-pair. However, since multiple test cases in the benchmark data may contain sequences of the same families, the samples independency assumption of the statistical test might not be fully met and for this reason the reported results should be interpreted with caution.

### BRAliBase 2.1

**Figure 2:**
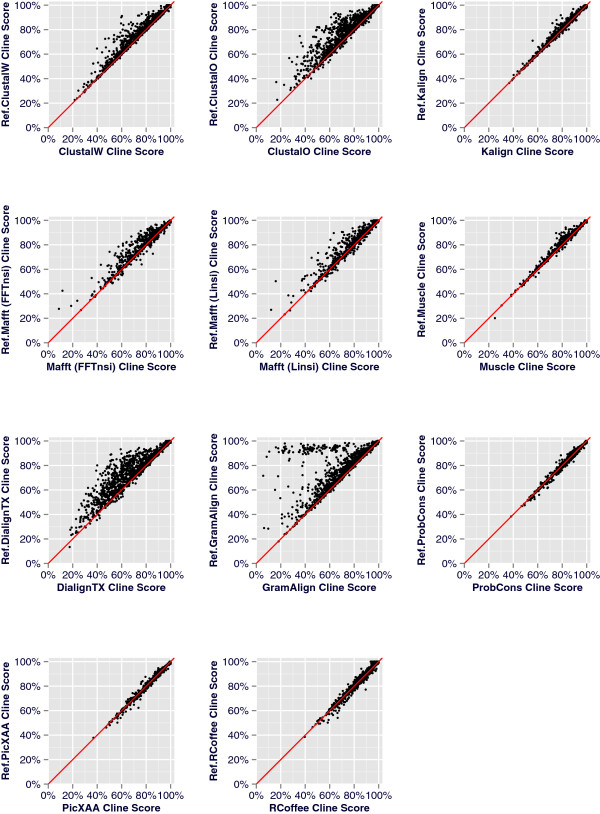
**Scatterplots of the Cline scores per alignment pair for the 2,218 benchmark tests of the BRAliBase dataset.** A scatterplot is constructed per alignment pair for each one of the 2,218 benchmark tests. The diagonal line represents the expected plot if the initial and the reformed alignments were identical. Consequently, an accumulation of points above the diagonal line represents improved reformed alignments whereas points below the diagonal correspond to cases where ReformAlign worsened the initial alignment.

**Figure 3:**
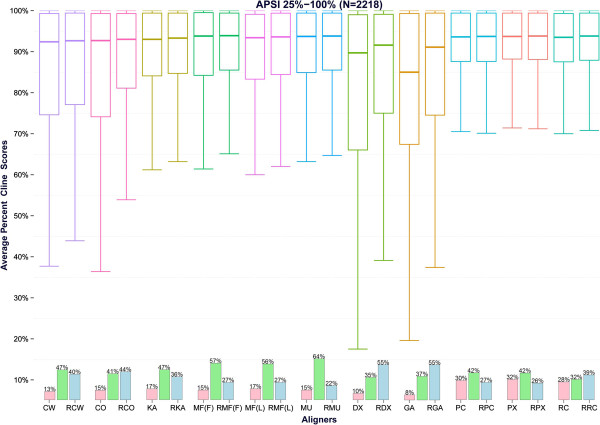
**Boxplots of the averaged Cline scores of the considered alignment pairs for the 2,218 alignments of the BRAliBase dataset.** The boxplots of the Cline scores averaged over all 2,218 test cases of the BRAliBase dataset are given at this figure. The figures are organized in pairs corresponding, from left to right, to the averaged performance of [ClustalW – Ref. ClustalW], [ClustalO – Ref. ClustalO], [Kalign – Ref. Kalign], [Mafft (FFTnsi) – Ref. Mafft (FFTnsi)], [Mafft (Linsi) – Ref.Mafft (Linsi)], [Muscle – Ref. Muscle], [DialignTX- Ref. DialignTX], [GramAlign – Ref. GramAlign], [ProbConsRNA – Ref. ProbConsRNA], [PicXAA – Ref. PicXAA], and [RCoffee – Ref. RCoffee]. The bars at the bottom of each pair represent the percentage of times where the reformed alignments were superior (blue bars), equal (green bars) or inferior (red bars) in terms of Cline score compared to the initial alignments.

**Figure 4:**
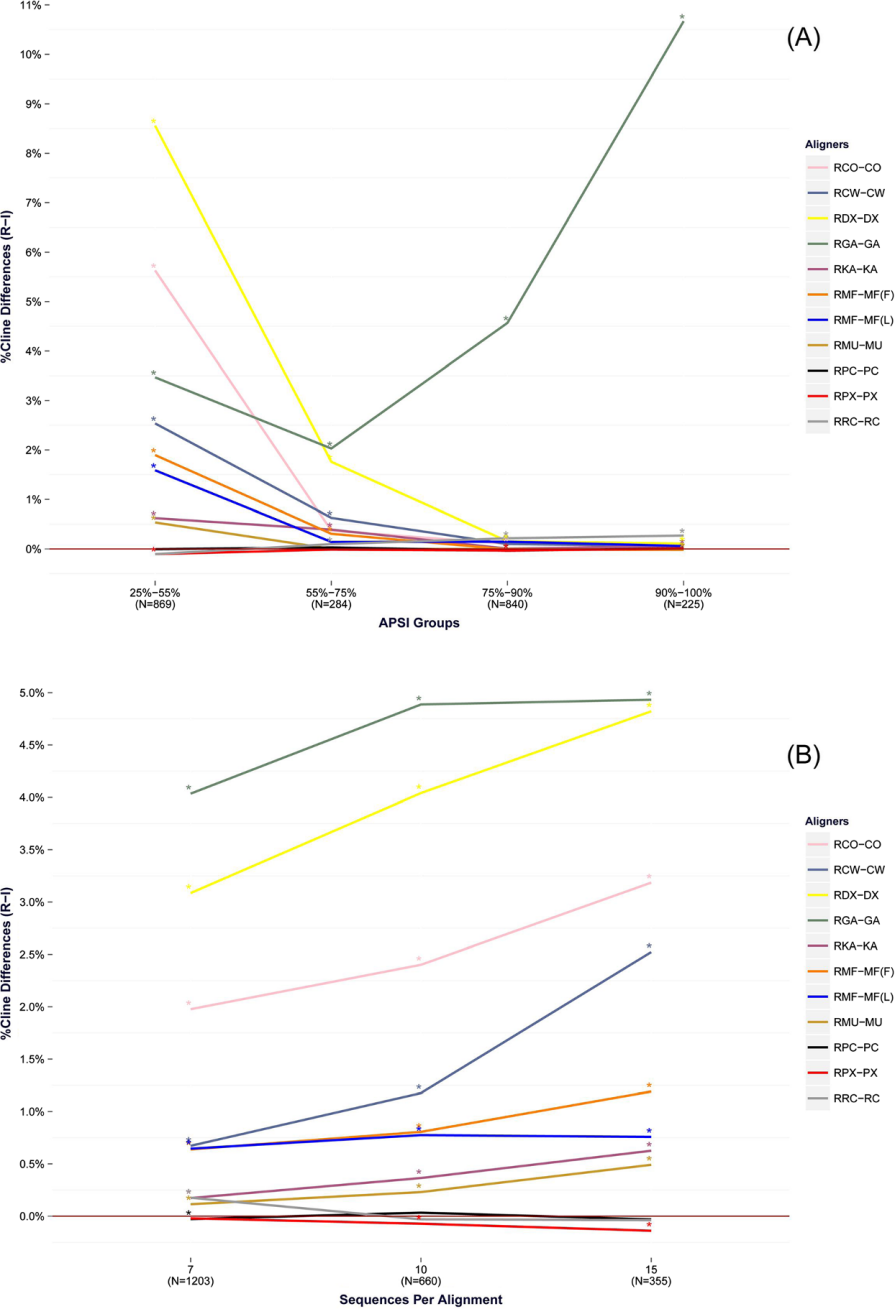
**Average Cline score differences for the benchmark tests of the BRAliBase dataset.** Effect of ReformAlign to testsets of increasing APSI **(A)** and increasing number of sequences per alignment **(B)**. The alignment pairs (from top to bottom, as appearing in the legends) correspond to [Ref. ClustalO – ClustalO], [Ref. ClustalW – ClustalW], [Ref. DialignTX – DialignTX], [Ref. GramAlign – GramAlign], [Ref. Kalign – Kalign], [Ref. Mafft (FFTnsi) – Mafft (FFTnsi)], [Ref. Mafft (Linsi) – Mafft (Linsi)], [Ref. Muscle – Muscle], [Ref. ProbConsRNA – ProbConsRNA], [Ref. PicXAA – PicXAA], and [Ref. RCoffee – RCoffee]. For each value of the horizontal axis, the difference is calculated by subtracting the average Cline score of the reformed alignments to the respective score of the initial alignments that belong to each group. Statistically significant differences at the .05 significance level are denoted by an asterisk (*) at the top-left corner of the respective points. The number of test cases (N) per group is given at the bottom of each value in the x-axis.

The results for the 2,218 BRAliBase benchmark tests are visualized in Figures 2 and 3. Moreover, in order to explore the effect of ReformAlign to testsets of increasing APSI, we grouped the benchmarks in four clusters according to the percent identity of the sequences. The average scores for each group are shown in Table 1 and Additional file 2: Table S2 and are graphically depicted at Figure 4.

**Table 1:** **BRAliBase Cline scores**

APSI [25%-100%] (N = 2,218)							
**Aligner**	**Initial (I)**	**Reformed (R)**	**Avg. Dif. (R-I)**	**p-value**	**R > I**	**R = I**	**R < I**
ClustalW	84.97%	86.09%	1.12%	p < 0.001***	40.22%	46.71%	13.07%
ClustalO	85.41%	87.71%	2.30%	p < 0.001***	44.00%	40.76%	15.24%
Kalign	89.30%	89.61%	0.30%	p < 0.001***	36.29%	46.93%	16.77%
Mafft (FFTnsi)	89.16%	89.94%	0.78%	p < 0.001***	27.23%	57.35%	15.42%
Mafft (Linsi)	88.63%	89.33%	0.70%	p < 0.001***	27.46%	55.91%	16.64%
Muscle	89.68%	89.89%	0.21%	p < 0.001***	21.51%	63.53%	14.97%
DialignTX	81.17%	84.82%	3.65%	p < 0.001***	54.78%	35.21%	10.01%
GramAlign	80.47%	84.90%	4.43%	p < 0.001***	54.64%	37.29%	8.07%
ProbCons	91.35%	91.34%	−0.01%	p = 0.127	27.41%	42.34%	30.25%
PicXAA	91.51%	91.46%	−0.06%	p < 0.001***	26.28%	41.88%	31.83%
R-Coffee	91.34%	91.42%	0.08%	p < 0.001***	39.18%	32.33%	28.49%
**APSI [25%-55%] (N = 869)**							
**Aligner**	**Initial (I)**	**Reformed (R)**	**Avg. Dif. (R-I)**	**p-value**	**R > I**	**R = I**	**R < I**
ClustalW	69.03%	71.57%	2.54%	p < 0.001***	71.00%	14.04%	14.96%
ClustalO	69.19%	74.82%	5.63%	p < 0.001***	79.29%	3.68%	17.03%
Kalign	79.32%	79.95%	0.63%	p < 0.001***	53.28%	22.09%	24.63%
Mafft (FFTnsi)	78.46%	80.35%	1.89%	p < 0.001***	53.51%	23.82%	22.67%
Mafft (Linsi)	77.79%	79.38%	1.59%	p < 0.001***	48.10%	26.12%	25.78%
Muscle	79.79%	80.33%	0.54%	p < 0.001***	42.58%	34.52%	22.90%
DialignTX	59.75%	68.31%	8.56%	p < 0.001***	90.33%	0.69%	8.98%
GramAlign	66.63%	70.10%	3.47%	p < 0.001***	78.02%	11.85%	10.13%
ProbCons	83.39%	83.39%	0.00%	p = 0.581	42.58%	10.01%	47.41%
PicXAA	83.77%	83.67%	−0.10%	p = 0.001***	40.05%	9.21%	50.75%
R-Coffee	83.67%	83.57%	−0.10%	p = 0.429	46.49%	4.83%	48.68%
**APSI [55%-75%] (N = 284)**							
**Aligner**	**Initial (I)**	**Reformed (R)**	**Avg. Dif. (R-I)**	**p-value**	**R > I**	**R = I**	**R < I**
ClustalW	84.78%	85.40%	0.62%	p < 0.001***	48.94%	25.35%	25.70%
ClustalO	87.53%	87.91%	0.38%	p = 0.001***	49.30%	11.62%	39.08%
Kalign	86.37%	86.76%	0.39%	p < 0.001***	55.28%	25.35%	19.37%
Mafft (FFTnsi)	87.46%	87.76%	0.30%	p = 0.004**	34.51%	37.32%	28.17%
Mafft (Linsi)	86.65%	86.80%	0.15%	p = 0.875	28.87%	39.08%	32.04%
Muscle	87.80%	87.80%	0.00%	p = 0.905	25.35%	46.48%	28.17%
DialignTX	83.49%	85.25%	1.76%	p < 0.001***	73.59%	5.63%	20.77%
GramAlign	81.75%	83.78%	2.03%	p < 0.001***	73.94%	17.25%	8.80%
ProbCons	89.23%	89.26%	0.03%	p = 0.686	41.20%	19.72%	39.08%
PicXAA	89.34%	89.33%	−0.01%	p = 0.609	39.08%	21.83%	39.08%
R-Coffee	89.49%	89.59%	0.10%	p = 0.014*	48.94%	11.27%	39.79%
**APSI [75%-90%] (N = 840)**							
**Aligner**	**Initial (I)**	**Reformed (R)**	**Avg. Dif. (R-I)**	**p-value**	**R > I**	**R = I**	**R < I**
ClustalW	98.16%	98.26%	0.10%	p < 0.001***	12.02%	81.79%	6.19%
ClustalO	98.20%	98.30%	0.10%	p < 0.001***	13.69%	80.36%	5.95%
Kalign	98.40%	98.41%	0.01%	p = 0.074	13.21%	77.62%	9.17%
Mafft (FFTnsi)	98.52%	98.51%	−0.01%	p = 0.178	3.93%	90.95%	5.12%
Mafft (Linsi)	98.08%	98.23%	0.15%	p < 0.001***	10.83%	85.60%	3.57%
Muscle	98.41%	98.41%	0.00%	p = 0.489	3.57%	92.38%	4.05%
DialignTX	98.15%	98.30%	0.15%	p < 0.001***	20.24%	72.86%	6.90%
GramAlign	92.78%	97.35%	4.57%	p < 0.001***	27.14%	66.07%	6.79%
ProbCons	98.61%	98.57%	−0.04%	p < 0.001***	8.33%	78.81%	12.86%
PicXAA	98.61%	98.58%	−0.03%	p < 0.001***	8.93%	77.62%	13.45%
R-Coffee	98.27%	98.48%	0.21%	p < 0.001***	27.74%	64.52%	7.74%
**APSI [95%-100%] (N = 225)**							
**Aligner**	**Initial (I)**	**Reformed (R)**	**Avg. Dif. (R-I)**	**p-value**	**R > I**	**R = I**	**R < I**
ClustalW	97.53%	97.59%	0.06%	p = 0.030*	15.56%	68.89%	15.56%
ClustalO	97.62%	97.66%	0.04%	p = 0.245	14.22%	72.89%	12.89%
Kalign	97.57%	97.63%	0.06%	p < 0.001***	32.89%	55.56%	11.56%
Mafft (FFTnsi)	97.72%	97.70%	−0.02%	p = 0.009**	3.56%	86.67%	9.78%
Mafft (Linsi)	97.67%	97.73%	0.06%	p = 0.642	8.00%	81.33%	10.67%
Muscle	97.68%	97.67%	−0.01%	p = 0.011*	2.22%	89.33%	8.44%
DialignTX	97.58%	97.69%	0.11%	p < 0.001***	22.67%	65.33%	12.00%
GramAlign	86.28%	96.95%	10.67%	p < 0.001***	42.67%	53.33%	4.00%
ProbCons	97.66%	97.67%	0.01%	p = 0.299	22.67%	59.56%	17.78%
PicXAA	97.67%	97.68%	0.01%	p = 0.526	21.78%	60.00%	18.22%
R-Coffee	97.41%	97.68%	0.27%	p < 0.001***	41.33%	44.89%	13.78%

Cline scores corresponding to the 2,218 alignments of the BRAliBase dataset. *significant at p<0.05, **significant at p<0.005, ***significant at p≤0.001. The first sub-table summarizes the performance of each alignment pair averaged over all test cases whereas the remaining sub-tables report the results organized according to the average pairwise sequence identity of the sequences. For every sub-table, the APSI value and the number of alignments (N) corresponding to each group are reported in the first row. Columns 2 and 3 correspond to the average Cline scores of the initial (I) and the reformed (R) alignments respectively. The average differences (R-I) per alignment pair are given in the fourth column and the corresponding p-values of these differences are given at the fifth column (statistically significant values at the .05 significance level are highlighted in bold). Finally, columns 6–8 indicate the percentage of cases where the reformed alignments were superior, equal or inferior (in terms of Cline score) to the initial alignment.

Despite the simplicity of the ReformAlign logic, the experimental results suggest that the proposed approach may significantly improve on the performance of almost all examined aligners. In general, the improvement due to ReformAlign seems to be substantially higher for difficult examples (i.e. alignments with relatively low APSI) or for lower-scoring aligners (e.g. DialignTX or GramAlign) and becomes less noticeable when the starting alignments are already nearly optimal leaving thus less room for improvement (Table 1, Additional file 2: Table S2 and Figure 4). The only cases where ReformAlign seems to degrade on average the initial alignments are for PicXAA and ProbConsRNA. Nonetheless, even for these aligners it was found that in a considerable amount of cases the reformed alignments were superior to the initial ones (Table 1 and Figure 3) whereas it may also be observed that the average decrease for both aligners is relatively small and not always statistically significant (Table 1). In addition, although the Cline (Table 1), SP and TC scores (Additional file 2: Table S2) seem to indicate a marginal degradation of the initial alignments due to ReformAlign, according to the D-POS similarity metric the reformed alignments were superior to the starting ones for all aligners participating in our experiments (Additional file 2: Table S2).

Another important observation is that ReformAlign very often delivers alignments which are different to the starting ones (Figure 3 and Table 1), and in the majority of these cases the reformed alignments are superior to the corresponding starting ones. This trend is much more apparent for testsets with relatively low APSI values and becomes less evident for highly similar alignment cases (Table 1 and Additional file 2: Table S2).

The experimental results also demonstrate that the application of ReformAlign to existing alignments does not affect all aligners in the same way and may result in changes in their overall ranking. For example, as it may be seen at Table 1, although initially GramAlign and R-Coffee score on average lower than DialignTX and ProbConsRNA respectively, the situation is reversed after ReformAlign has been applied to fine-tune the respective alignments. The same holds true for the MUSCLE, Kalign and Mafft (with the FFT-NS-i setting) aligners with the latter scoring higher than the other two after the respective alignments have been reformed.

Finally, as it may be seen at Figure 4A, the effect of ReformAlign for the majority of aligners seems to be more substantial for harder test cases (APSI ≤ 75%) and becomes less noticeable as the average pairwise sequence identity increases. This does not seem to be the case for the highest scoring aligners (ProbConsRNA, PicXAA and R-Coffee) which deliver quite accurate alignments (Table 1 and Additional file 2: Table S2) even for relatively hard test cases leaving thus less room for improvement, and for GramAlign where several test cases from the higher APSI groups seem to violate its grammar-based assumptions resulting thus in lower-scoring initial alignments that are then refined by ReformAlign (Figures 3 and 4, Table 1 and Additional file 2: Table S2).

The analysis of the Cline score differences versus the number of sequences per alignment (Figure 4B) revealed similar results. Probabilistic and consistency-based aligners, which appear to deliver more accurate starting alignments, seem to benefit less (or are even marginally worsened) from the application of ReformAlign compared to optimization-driven or iterative refinement based approaches. The main reason for this might be in the underlying assumptions of these models. In particular, these high-scoring aligners employ sophisticated albeit computationally expensive probabilistic assumptions resulting thus quite often in very accurate alignments. ReformAlign however is based on the *ad hoc* SP scoring scheme and it may thus happen that accurate starting alignments that meet the probabilistic/consistency based assumptions are slightly degraded by the more arbitrary optimization scheme of ReformAlign, especially in alignments composed of multiple sequences. However, as the experimental results demonstrate, there is a considerable amount of cases where the initial alignments of the Probabilistic Consistency Transformation (PCT) approaches are improved by the proposed post-processing step (Table 1, Figures 2 and 3), suggesting thus that ReformAlign could appear to be useful even for fine-tuning the starting alignments of such sophisticated alignment methods.

### DNA SMART

**Figure 5:**
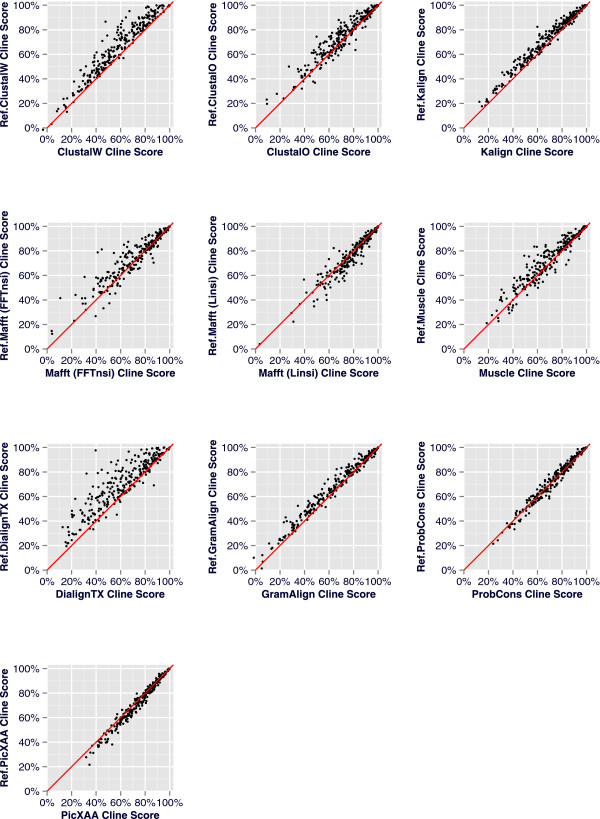
**Scatterplots of the Cline scores per alignment pair for the 264 benchmark tests of the DNA SMART dataset.** For each alignment pair a scatterplot is constructed. Each point corresponds to an alignment test case of the DNA SMART dataset and depicts the Cline score of the initial alignment versus the respective score of the reformed alignment. The diagonal line represents the expected plot if the initial and the reformed alignments were identical.

**Figure 6:**
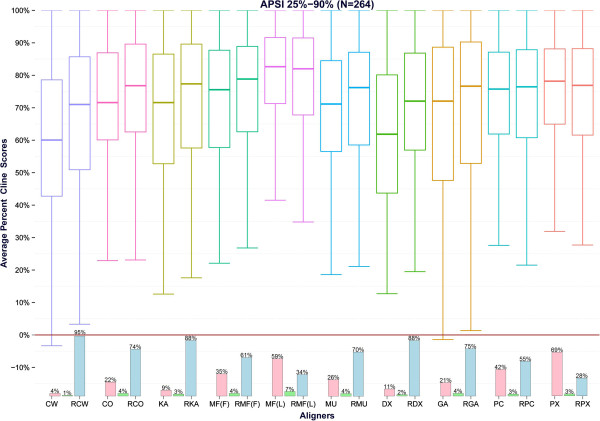
**Boxplots of the averaged Cline scores for the 264 alignments of the DNA SMART dataset.** The boxplots corresponding to the Cline scores for the DNA SMART dataset are given in this figure. The figures, correspond, from left to right, to the averaged performance of [ClustalW – Ref. ClustalW], [ClustalO – Ref. ClustalO], [Kalign – Ref. Kalign], [Mafft (FFTnsi) – Ref. Mafft (FFTnsi)], [Mafft (Linsi) – Ref.Mafft (Linsi)], [Muscle – Ref. Muscle], [DialignTX- Ref. DialignTX], [GramAlign – Ref. GramAlign], [ProbConsRNA – Ref. ProbConsRNA] and [PicXAA – Ref. PicXAA]. The bars at the bottom of each pair represent the percentage of times where the reformed alignments were superior (blue bars), equal (green bars) or inferior (red bars) in terms of Cline score compared to the initial alignments.

A similar analysis was carried out for the benchmark tests of the DNA SMART database. For these experiments R-Coffee (which is specialized for RNA alignments) was removed from the analysis. Since the majority of the test cases (257 out of 264 alignments) were sharing an average sequence identity equal or lower to 55%, the differences between the reformed and the initial alignments for the higher APSI groups were not found to be statistically significant at the .05 level (data not shown). The performance averaged over all 264 test cases per alignment pair is graphically depicted at Figures 5 and 6 whereas the analytic results for the considered accuracy metrics are reported at Table 2 and Additional file 3: Table S3.

**Table 2:** **DNA SMART Cline scores**

APSI [25%-90%] (N = 264)							
**Aligner**	**Initial (I)**	**Reformed (R)**	**Avg. Dif. (R-I)**	**p-value**	**R > I**	**R = I**	**R < I**
ClustalW	59.55%	67.06%	7.51%	p < 0.001***	95.08%	0.76%	4.17%
ClustalO	70.94%	74.25%	3.31%	p < 0.001***	74.24%	3.79%	21.97%
Kalign	68.37%	72.37%	4.00%	p < 0.001***	87.50%	3.41%	9.09%
Mafft (FFTnsi)	71.93%	74.81%	2.88%	p < 0.001***	60.98%	3.79%	35.23%
Mafft (Linsi)	79.70%	78.26%	−1.44%	p < 0.001***	33.71%	7.20%	59.09%
Muscle	69.51%	72.52%	3.01%	p < 0.001***	70.08%	3.79%	26.14%
DialignTX	60.82%	70.13%	9.31%	p < 0.001***	87.50%	1.52%	10.98%
GramAlign	66.96%	70.11%	3.15%	p < 0.001***	74.62%	4.17%	21.21%
ProbCons	73.68%	73.90%	0.22%	p = 0.099	54.55%	3.41%	42.05%
PicXAA	75.64%	73.85%	−1.79%	p < 0.001***	28.03%	3.03%	68.94%

Cline scores corresponding to the 264 alignments of the DNA SMART dataset. *significant at p<0.05, **significant at p<0.005, ***significant at p≤0.001. The average Cline scores corresponding to the initial (I) and the reformed (R) alignments are given at columns 2 and 3 respectively. Column 4 represents the differences of the Cline Scores (R-I) per alignment pair and the statistical significance of these differences is given at column 5 (statistically significant values at the .05 significance level are highlighted in bold). The last three columns correspond to the percentage of cases where the reformed alignments were superior, equal or inferior (in terms of Cline score) to the initial alignment.

The experimental results suggest that ReformAlign was able to improve on the starting alignments for the majority of the considered aligners. For ProbConsRNA the effect of ReformAlign is dubious: although on average the reformed alignments appear to be marginally superior to the initial ones (Table 2 and Additional file 3: Table S3), they demonstrate a greater variability compared to the starting alignments (Figure 6) and the reported differences are not always found to be statistically significant. Regarding the remaining aligners of our benchmark study, only Mafft (with the L-NS-i setting) and PicXAA are shown to be negatively affected by the application of ReformAlign whereas a noticeable improvement is observed for the rest. The reported degradation of the PCT-based aligners could be due to the sophisticated assumptions of these high performance methods compared to the *ad hoc* optimization scheme of ReformAlign. Specifically, since the DNA SMART database is composed of alignments which are based on biological features such as the tertiary structure of encoded proteins, the simplicity of the ReformAlign scheme could result more often in degradation of alignments deriving from the more accurate PCT-based methods, compared to alignments generated from progressive and iterative refinement aligners.

In agreement to the results of the BRAliBase experiments, the DNA SMART analysis supports the conclusions that ReformAlign generates alignments that are frequently different (and often superior) to the starting ones (Figure 6 and Table 2) and that the suggested approach does not affect all aligners the same way, resulting thus in changes in their overall ranking (Table 2 and Additional file 3: Table S3).

**Figure 7:**
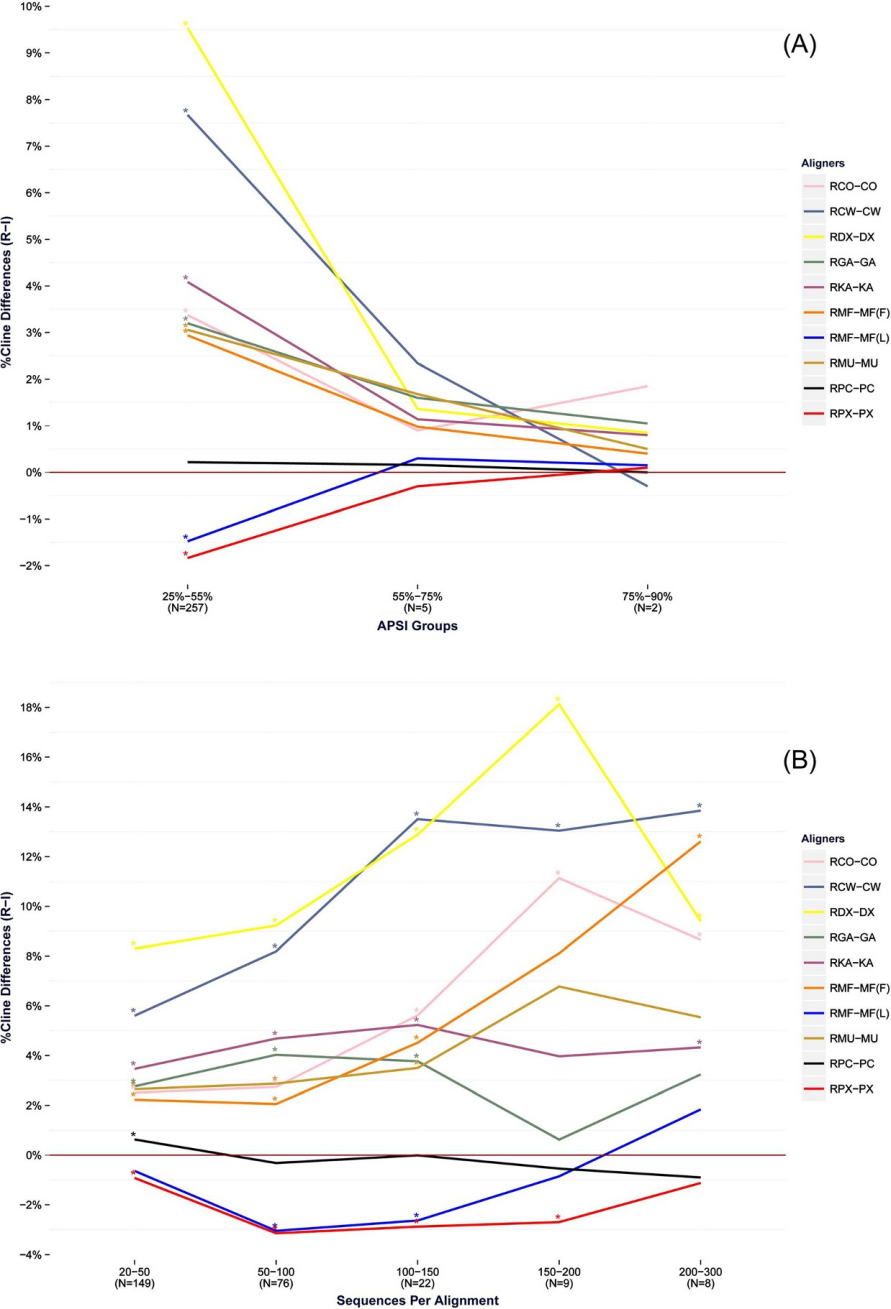
**Average Cline score differences for the benchmark tests of the DNA SMART dataset.** Effect of ReformAlign to testsets of increasing APSI **(A)** and increasing number of sequences per alignment **(B)**. The alignment pairs (from top to bottom, as appearing in the legends) correspond to [Ref. ClustalO – ClustalO], [Ref. ClustalW – ClustalW], [Ref. DialignTX – DialignTX], [Ref. GramAlign – GramAlign], [Ref. Kalign – Kalign], [Ref. Mafft (FFTnsi) – Mafft (FFTnsi)], [Ref. Mafft (Linsi) – Mafft (Linsi)], [Ref. Muscle – Muscle], [Ref. ProbConsRNA – ProbConsRNA] and [Ref. PicXAA – PicXAA]. For each value of the horizontal axis, the difference is calculated by subtracting the average Cline score of the reformed alignments to the respective score of the initial alignments that belong to each group. Statistically significant differences at the .05 significance level are denoted by an asterisk (*) at the top-left corner of the respective points.

Finally, the assessment of the effect of ReformAlign to testsets of increasing APSI (Figure 7A) and increasing number of sequences per alignment (Figure 7B) did not provide us with conclusive results, mainly due to the fact that there were only a limited number of test cases belonging to the higher order groups, often resulting in statistically insignificant differences for the considered alignment pairs. Nevertheless, the results of Figure 7A indicate a trend that further supports the idea that the effect of ReformAlign is weaker for closely related sequences compared to alignments with lower APSI values, whereas there does not seem to be a clear pattern describing the way ReformAlign affects the examined aligners with an increasing number of sequences per alignment (Figure 7B).

## Conclusion

In this paper we presented ReformAlign, a novel profile-based meta-alignment approach that aims at correcting early alignment errors by giving the sequences a second opportunity to re-align themselves against a standard profile that efficiently summarizes the initial alignment information. ReformAlign is based on the Gotoh’s affine-gap penalties variation of the classic Needleman-Wunsch algorithm and uses a refinement scheme according to which the reformed alignment is indirectly inferred by a series of individual and independent sequence-profile pairwise comparisons.

ReformAlign was extensively assessed on the way it affects the alignments of ten leading aligners using benchmark testsets from the BRAliBase and DNA SMART datasets. The results suggest that the majority of aligners showed a notable improvement on the accuracy of the delivered alignments when ReformAlign was employed as a post-processing step. This improvement was more substantial for harder alignment cases with low APSI or when there was still adequate room for improvement on the starting alignments, and became less evident when the initial alignment was already quite accurate or for easier alignment cases with high identity rates.

Finally, it is important to mention that the proposed method does not come to replace other popular alignment techniques. Instead, users may continue to use their aligner(s) of preference (even programs that were not examined in the present study) and then complementarily employ ReformAlign as a post-processing step to examine if the delivered alignment is more appropriate for their analyses.

## Availability and requirements

ReformAlign is freely available to the public under the GNU General Public License (version 3 or later). Both the source code and precompiled binaries for Linux and Windows may be downloaded from http://evol.bio.lmu.de/_statgen/software/reformalign/. Currently ReformAlign can only align DNA and RNA sequences and it is provided as a command line program. Its source code is in C++ and it makes use of the Open Multi-Processing (OpenMP) parallelization API.

For the benchmark tests, the BRAliBase 2.1 RNA alignment database [28] and the alignments from the DNA Reference Alignment Benchmarks database [29] were employed. The evaluation of the alignments accuracy was performed using Robert Edgar’s QScore, (v.2.1) multiple alignment scoring software and Blackburne and Whelan’s MetAl (v.1.1) [37] command-line utility for calculating metric distances between alternative alignments.

Finally, the alignment programs that were used in our benchmark analysis are as follows: ClustalW2 [38] (v.2.1), ClustalO [39] (v.1.2), MUSCLE [8, 26] (v.3.8.31), MAFFT [40] (v.7.149), Kalign [7] (v.2.04), GramAlign [41] (v.3), ProbConsRNA [42] (v.1.1), R-Coffee [43, 44] (T-Coffee v.10.00.r1613), PicXAA [45] (v.1.03) and Dialign-TX [46] (v.1.0.2).

## Electronic supplementary material

Additional file 1: Table S1: Command-line options per alignment method. (DOC 30 KB)

Additional file 2: Table S2: BRAliBase Results. Averaged D-POS, SP and TC scores for the 2,218 benchmark tests of the BRAliBase dataset. The results are presented averaged over all benchmark tests as well as organized in four groups according to the average pairwise sequence identity of the sequences. For each accuracy metric three figures are provided corresponding to the average scores of the initial alignments (I), the reformed alignments (R) and their respective differences (R-I). Differences that are found to be statistically significant at the .05 significance level are highlighted in bold. For the SP and TC scores, positive average differences indicate that the reformed alignments were superior to the initial ones whereas negative values indicate that ReformAlign (on average) worsened the starting alignments. For the D-POS scores the situation is reversed: negative values indicate an improvement due to ReformAlign, whereas positive values represent a degradation of the starting alignments. (DOC 125 KB)

Additional file 3: Table S3: DNA SMART Results. Averaged D-POS, SP and TC scores for the 264 benchmark tests of the DNA SMART dataset. For each accuracy metric three figures are provided corresponding to the average scores of the initial alignments (I), the reformed alignments (R) and their respective differences (R-I). Statistically significant differences at the .05 significance level are highlighted in bold. (DOC 42 KB)

## References

[1] Notredame C (2007). Recent evolutions of multiple sequence alignment algorithms. PLoS Comput Biol.

[2] Edgar RC, Batzoglou S (2006). Multiple sequence alignment. Curr Opin Struct Biol.

[3] Notredame C (2002). Recent progress in multiple sequence alignment: a survey. Pharmacogenomics.

[4] Do CB, Katoh K (2008). Protein multiple sequence alignment. Methods Mol Biol Clifton NJ.

[5] Murata M, Richardson JS, Sussman JL (1985). Simultaneous comparison of three protein sequences. Proc Natl Acad Sci U S A.

[6] Thompson JD, Higgins DG, Gibson TJ (1994). CLUSTAL W: improving the sensitivity of progressive multiple sequence alignment through sequence weighting, position-specific gap penalties and weight matrix choice. Nucleic Acids Res.

[7] Lassmann T, Frings O, Sonnhammer ELL (2009). Kalign2: high-performance multiple alignment of protein and nucleotide sequences allowing external features. Nucleic Acids Res.

[8] Edgar RC (2004). MUSCLE: multiple sequence alignment with high accuracy and high throughput. Nucleic Acids Res.

[9] Just W (2001). Computational complexity of multiple sequence alignment with SP-score. J Comput Biol J Comput Mol Cell Biol.

[10] Kececioglu J, Starrett D (2004). Aligning alignments exactly. Proc Eighth Annu Int Conf Res Comput Mol Biol.

[11] Wang L, Jiang T (1994). On the complexity of multiple sequence alignment. J Comput Biol J Comput Mol Cell Biol.

[12] Bonizzoni P, Vedova GD (2001). The complexity of multiple sequence alignment with SP-score that is a metric. Theor Comput Sci.

[13] Feng DF, Doolittle RF (1987). Progressive sequence alignment as a prerequisite to correct phylogenetic trees. J Mol Evol.

[14] Hogeweg P, Hesper B (1984). The alignment of sets of sequences and the construction of phyletic trees: an integrated method. J Mol Evol.

[15] Barton GJ, Sternberg MJ (1987). A strategy for the rapid multiple alignment of protein sequences. Confidence levels from tertiary structure comparisons. J Mol Biol.

[16] Higgins DG, Sharp PM (1989). Fast and sensitive multiple sequence alignments on a microcomputer. Comput Appl Biosci CABIOS.

[17] Durbin R, Eddy S, Krogh A, Mitchison G (1998). Biological Sequence Analysis: Probabilistic Models of Proteins and Nucleic Acids.

[18] Berger MP, Munson PJ (1991). A novel randomized iterative strategy for aligning multiple protein sequences. Comput Appl Biosci CABIOS.

[19] Gotoh O (1993). Optimal alignment between groups of sequences and its application to multiple sequence alignment. Comput Appl Biosci CABIOS.

[20] Roskin KM, Paten B, Haussler D (2011). Meta-alignment with crumble and prune: partitioning very large alignment problems for performance and parallelization. BMC Bioinformatics.

[21] Gotoh O (1982). An improved algorithm for matching biological sequences. J Mol Biol.

[22] Needleman SB, Wunsch CD (1970). A general method applicable to the search for similarities in the amino acid sequence of two proteins. J Mol Biol.

[23] Ye X, Wang G, Altschul SF (2011). An assessment of substitution scores for protein profile-profile comparison. Bioinformatics.

[24] Edgar RC (2009). Optimizing substitution matrix choice and gap parameters for sequence alignment. BMC Bioinformatics.

[25] Chiaromonte F, Yap VB, Miller W (2002). Scoring pairwise genomic sequence alignments. Pac Symp Biocomput Pac Symp Biocomput.

[26] Edgar RC (2004). MUSCLE: a multiple sequence alignment method with reduced time and space complexity. BMC Bioinformatics.

[27] Gardner PP, Wilm A, Washietl S (2005). A benchmark of multiple sequence alignment programs upon structural RNAs. Nucleic Acids Res.

[28] Wilm A, Mainz I, Steger G (2006). An enhanced RNA alignment benchmark for sequence alignment programs. Algorithms Mol Biol.

[29] Carroll H, Beckstead W, O’Connor T, Ebbert M, Clement M, Snell Q, McClellan D (2007). DNA reference alignment benchmarks based on tertiary structure of encoded proteins. Bioinformatics.

[30] Gardner PP, Giegerich R (2004). A comprehensive comparison of comparative RNA structure prediction approaches. BMC Bioinformatics.

[31] Thompson JD, Koehl P, Ripp R, Poch O (2005). BAliBASE 3.0: Latest developments of the multiple sequence alignment benchmark. Proteins Struct Funct Bioinforma.

[32] Raghava GPS, Searle SM, Audley PC, Barber JD, Barton GJ (2003). OXBench: A benchmark for evaluation of protein multiple sequence alignment accuracy. BMC Bioinformatics.

[33] Ponting CP, Schultz J, Milpetz F, Bork P (1999). SMART: identification and annotation of domains from signalling and extracellular protein sequences. Nucleic Acids Res.

[34] Sauder JM, Arthur JW, Dunbrack RL (2000). Large-scale comparison of protein sequence alignment algorithms with structure alignments. Proteins.

[35] Thompson JD, Plewniak F, Poch O (1999). A comprehensive comparison of multiple sequence alignment programs. Nucleic Acids Res.

[36] Cline M, Hughey R, Karplus K (2002). Predicting reliable regions in protein sequence alignments. Bioinformatics.

[37] Blackburne BP, Whelan S (2012). Measuring the distance between multiple sequence alignments. Bioinformatics.

[38] Larkin MA, Blackshields G, Brown NP, Chenna R, McGettigan PA, McWilliam H, Valentin F, Wallace IM, Wilm A, Lopez R, Thompson JD, Gibson TJ, Higgins DG (2007). Clustal W and Clustal X version 2.0. Bioinformatics.

[39] Sievers F, Wilm A, Dineen D, Gibson TJ, Karplus K, Li W, Lopez R, McWilliam H, Remmert M, Söding J, Thompson JD, Higgins DG (2011). Fast, scalable generation of high‒quality protein multiple sequence alignments using Clustal Omega. Mol Syst Biol.

[40] Katoh K, Standley DM (2013). MAFFT multiple sequence alignment software Version 7: improvements in performance and usability. Mol Biol Evol.

[41] Russell DJ, Way SF, Benson AK, Sayood K (2010). A grammar-based distance metric enables fast and accurate clustering of large sets of 16S sequences. BMC Bioinformatics.

[42] Do CB, Mahabhashyam MSP, Brudno M, Batzoglou S (2005). ProbCons: Probabilistic consistency-based multiple sequence alignment. Genome Res.

[43] Wilm A, Higgins DG, Notredame C (2008). R-Coffee: a method for multiple alignment of non-coding RNA. Nucleic Acids Res.

[44] Notredame C, Higgins DG, Heringa J (2000). T-Coffee: a novel method for fast and accurate multiple sequence alignment. J Mol Biol.

[45] Sahraeian SME, Yoon B-J (2010). PicXAA: greedy probabilistic construction of maximum expected accuracy alignment of multiple sequences. Nucleic Acids Res.

[46] Subramanian AR, Kaufmann M, Morgenstern B (2008). DIALIGN-TX: greedy and progressive approaches for segment-based multiple sequence alignment. Algorithms Mol Biol.

