# Supplementary material for: ReformAlign: improved multiple sequence alignments using a profile-based meta-alignment approach
Source: BMC Bioinformatics. 2014 Aug 7;15(1):265. doi: 10.1186/1471-2105-15-265 (PMC4133627; doi:10.1186/1471-2105-15-265)
Supplement: Supplementary file 1 — Additional file 1: Table S1: Command-line options per alignment method. (DOC 30 KB) [file 12859_2014_6534_MOESM1_ESM.doc]

## Additional file 1: Table S1 - Command-line options per alignment method

| **Aligner** | **Command** |
| --- | --- |
| ClustalW | clustalw2 <infile> -output=FASTA -outfile=<outfile> -outorder=INPUT -quiet |
| ClustalO | clustalo -i <infile> -o <outfile> |
| Kalign | kalign <infile> <outfile> |
| Mafft (FFTnsi) | mafft --retree 2 --maxiterate 1000 <infile> > <outfile> |
| Mafft (Linsi) | mafft --localpair --maxiterate 1000 <infile> > <outfile> |
| Muscle | muscle -in <infile> -out <outfile> -quiet |
| DialignTX | dialign-tx <conf –directory> <infile> <outfile> |
| GramAlign | gramalign -i <infile> -o <outfile> -f 2 |
| ProbConsRNA | probcons <infile> > <outfile> |
| PicXAA | picxaa -PHMM -nuc <infile> > <outfile> |
| R-Coffee | t_coffee <infile> -mode rcoffee > <outfile> |
| ReformAlign | reformalign -i <infile> -o <outfile> -a <initalignmentfile> |
