# Supplementary material for: ReformAlign: improved multiple sequence alignments using a profile-based meta-alignment approach
Source: BMC Bioinformatics. 2014 Aug 7;15(1):265. doi: 10.1186/1471-2105-15-265 (PMC4133627; doi:10.1186/1471-2105-15-265)
Supplement: Supplementary file 2 — Additional file 2: Table S2: BRAliBase Results. Averaged D-POS, SP and TC scores for the 2,218 benchmark tests of the BRAliBase dataset. The results are presented averaged over all benchmark tests as well as organized in four groups according to the average pairwise sequence identity of the sequences. For each accuracy metric three figures are provided corresponding to the average scores of the initial alignments (I), the reformed alignments (R) and their respective differences (R-I). Differences that are found to be statistically significant at the .05 significance level are highlighted in bold. For the SP and TC scores, positive average differences indicate that the reformed alignments were superior to the initial ones whereas negative values indicate that ReformAlign (on average) worsened the starting alignments. For the D-POS scores the situation is reversed: negative values indicate an improvement due to ReformAlign, whereas positive values represent a degradation of the starting alignments. (DOC 125 KB) [file 12859_2014_6534_MOESM2_ESM.doc]

## Additional file 2: Table S2 – BRAliBase Results

Averaged D-POS, SP and TC scores for the 2,218 benchmark tests of the BRAliBase dataset. The results are presented averaged over all benchmark tests as well as organized in four groups according to the average pairwise sequence identity of the sequences. For each accuracy metric three figures are provided corresponding to the average scores of the initial alignments (I), the reformed alignments (R) and their respective differences (R-I). Differences that are found to be statistically significant at the .05 significance level are highlighted in bold. For the SP and TC scores, positive average differences indicate that the reformed alignments were superior to the initial ones whereas negative values indicate that ReformAlign (on average) worsened the starting alignments. For the D-POS scores the situation is reversed: negative values indicate an improvement due to ReformAlign, whereas positive values represent a degradation of the starting alignments.

| **APSI [25%-100%] (N=2,218)** | | | | | | | | | |
| --- | --- | --- | --- | --- | --- | --- | --- | --- | --- |
|  | **D-POS** | | | **SP** | | | **TC** | | |
| **Aligner** | **(I)** | **(R)** | **(R-I)** | **(I)** | **(R)** | **(R-I)** | **(I)** | **(R)** | **(R-I)** |
| ClustalW | 19.40% | 18.17% | **-1.23%** | 82.78% | 84.09% | **1.31%** | 64.10% | 66.05% | **1.95%** |
| ClustalO | 19.05% | 16.81% | **-2.24%** | 83.19% | 85.55% | **2.36%** | 65.22% | 68.60% | **3.38%** |
| Kalign | 15.92% | 15.04% | **-0.88%** | 87.41% | 87.77% | **0.36%** | 70.89% | 71.65% | **0.76%** |
| Mafft (FFTnsi) | 14.90% | 14.25% | **-0.65%** | 87.55% | 88.23% | **0.68%** | 72.28% | 73.08% | **0.80%** |
| Mafft (Linsi) | 15.60% | 14.95% | **-0.65%** | 86.86% | 87.53% | **0.67%** | 70.20% | 71.51% | **1.31%** |
| Muscle | 14.55% | 14.36% | **-0.19%** | 87.90% | 88.12% | **0.22%** | 71.78% | 72.20% | **0.42%** |
| DialignTX | 23.84% | 20.19% | **-3.65%** | 78.21% | 82.04% | **3.83%** | 59.22% | 63.82% | **4.60%** |
| GramAlign | 23.71% | 19.84% | **-3.87%** | 78.16% | 82.47% | **4.31%** | 59.86% | 66.25% | **6.39%** |
| ProbCons | 13.50% | 13.23% | **-0.27%** | 89.69% | 89.69% | 0.00% | 74.64% | 74.62% | -0.02% |
| PicXAA | 13.27% | 13.09% | **-0.18%** | 89.88% | 89.82% | **-0.06%** | 74.90% | 74.79% | **-0.11%** |
| R-Coffee | 13.69% | 13.24% | **-0.45%** | 89.35% | 89.63% | **0.28%** | 73.45% | 74.26% | **0.81%** |

| **APSI [25%-55%] (N=869)** | | | | | | | | | |
| --- | --- | --- | --- | --- | --- | --- | --- | --- | --- |
|  | **D-POS** | | | **SP** | | | **TC** | | |
| **Aligner** | **(I)** | **(R)** | **(R-I)** | **(I)** | **(R)** | **(R-I)** | **(I)** | **(R)** | **(R-I)** |
| ClustalW | 38.45% | 35.69% | **-2.76%** | 64.50% | 67.43% | **2.93%** | 29.98% | 34.01% | **4.03%** |
| ClustalO | 38.58% | 33.16% | **-5.42%** | 64.46% | 70.16% | **5.70%** | 30.46% | 38.42% | **7.96%** |
| Kalign | 28.42% | 27.61% | **-0.81%** | 75.38% | 76.14% | **0.76%** | 45.69% | 47.12% | **1.43%** |
| Mafft (FFTnsi) | 28.41% | 26.85% | **-1.56%** | 75.17% | 76.82% | **1.65%** | 47.24% | 49.32% | **2.08%** |
| Mafft (Linsi) | 29.25% | 27.88% | **-1.37%** | 74.34% | 75.77% | **1.43%** | 44.48% | 46.93% | **2.45%** |
| Muscle | 27.45% | 26.95% | **-0.50%** | 76.15% | 76.68% | **0.53%** | 46.75% | 47.86% | **1.11%** |
| DialignTX | 48.91% | 40.52% | **-8.39%** | 53.68% | 62.51% | **8.83%** | 19.10% | 28.63% | **9.53%** |
| GramAlign | 42.11% | 38.47% | **-3.64%** | 60.99% | 64.81% | **3.82%** | 32.43% | 36.74% | **4.31%** |
| ProbCons | 24.09% | 23.99% | -0.10% | 79.90% | 79.94% | 0.04% | 52.12% | 52.17% | 0.05% |
| PicXAA | 23.65% | 23.68% | 0.03% | 80.34% | 80.25% | **-0.09%** | 52.68% | 52.53% | -0.15% |
| R-Coffee | 24.25% | 24.05% | **-0.20%** | 79.65% | 79.84% | **0.19%** | 50.97% | 51.51% | **0.54%** |

| **APSI [55%-75%] (N=284)** | | | | | | | | | |
| --- | --- | --- | --- | --- | --- | --- | --- | --- | --- |
|  | **D-POS** | | | **SP** | | | **TC** | | |
| **Aligner** | **(I)** | **(R)** | **(R-I)** | **(I)** | **(R)** | **(R-I)** | **(I)** | **(R)** | **(R-I)** |
| ClustalW | 20.72% | 20.04% | **-0.68%** | 82.18% | 82.93% | **0.74%** | 59.27% | 60.41% | **1.14%** |
| ClustalO | 17.74% | 17.32% | **-0.42%** | 85.26% | 85.80% | **0.54%** | 65.47% | 65.91% | 0.44% |
| Kalign | 19.51% | 18.91% | **-0.60%** | 83.88% | 84.31% | **0.43%** | 60.82% | 61.83% | **1.01%** |
| Mafft (FFTnsi) | 18.11% | 17.79% | **-0.32%** | 85.01% | 85.34% | **0.33%** | 64.55% | 64.88% | 0.33% |
| Mafft (Linsi) | 18.96% | 18.83% | -0.13% | 84.11% | 84.28% | 0.17% | 63.09% | 63.09% | 0.00% |
| Muscle | 17.77% | 17.78% | 0.01% | 85.34% | 85.38% | 0.04% | 64.12% | 64.02% | -0.10% |
| DialignTX | 23.20% | 21.19% | **-2.01%** | 79.70% | 81.83% | **2.13%** | 54.19% | 58.47% | **4.28%** |
| GramAlign | 24.71% | 22.23% | **-2.48%** | 78.19% | 80.81% | **2.62%** | 52.21% | 57.73% | **5.52%** |
| ProbCons | 16.50% | 16.36% | **-0.14%** | 86.99% | 87.02% | 0.03% | 67.61% | 67.71% | 0.10% |
| PicXAA | 16.41% | 16.29% | -0.12% | 87.10% | 87.09% | -0.01% | 67.90% | 68.04% | 0.14% |
| R-Coffee | 16.35% | 16.02% | **-0.33%** | 87.03% | 87.30% | **0.27%** | 67.51% | 68.10% | **0.59%** |

| **APSI [75%-90%] (N=840)** | | | | | | | | | |
| --- | --- | --- | --- | --- | --- | --- | --- | --- | --- |
|  | **D-POS** | | | **SP** | | | **TC** | | |
| **Aligner** | **(I)** | **(R)** | **(R-I)** | **(I)** | **(R)** | **(R-I)** | **(I)** | **(R)** | **(R-I)** |
| ClustalW | 3.21% | 3.07% | **-0.14%** | 97.93% | 98.07% | **0.14%** | 93.52% | 93.98% | **0.46%** |
| ClustalO | 3.20% | 3.05% | **-0.15%** | 97.96% | 98.10% | **0.14%** | 93.68% | 94.14% | **0.46%** |
| Kalign | 3.89% | 3.25% | **-0.64%** | 98.30% | 98.30% | 0.00% | 94.52% | 94.60% | **0.08%** |
| Mafft (FFTnsi) | 2.67% | 2.69% | 0.02% | 98.44% | 98.42% | -0.02% | 95.13% | 95.00% | **-0.13%** |
| Mafft (Linsi) | 3.34% | 3.12% | **-0.22%** | 97.82% | 98.03% | **0.21%** | 93.14% | 93.94% | **0.80%** |
| Muscle | 2.85% | 2.85% | 0.00% | 98.28% | 98.28% | 0.00% | 94.54% | 94.54% | 0.00% |
| DialignTX | 3.29% | 3.07% | **-0.22%** | 97.88% | 98.09% | **0.21%** | 93.48% | 94.17% | **0.69%** |
| GramAlign | 7.23% | 3.72% | **-3.51%** | 93.27% | 97.36% | **4.09%** | 86.58% | 92.82% | **6.24%** |
| ProbCons | 3.32% | 3.08% | **-0.24%** | 98.55% | 98.50% | **-0.05%** | 95.35% | 95.16% | **-0.19%** |
| PicXAA | 3.27% | 3.06% | **-0.21%** | 98.55% | 98.50% | **-0.05%** | 95.34% | 95.14% | **-0.20%** |
| R-Coffee | 3.75% | 3.19% | **-0.56%** | 98.00% | 98.36% | **0.36%** | 93.75% | 94.76% | **1.01%** |

| **APSI [90%-100%] (N=225)** | | | | | | | | | |
| --- | --- | --- | --- | --- | --- | --- | --- | --- | --- |
|  | **D-POS** | | | **SP** | | | **TC** | | |
| **Aligner** | **(I)** | **(R)** | **(R-I)** | **(I)** | **(R)** | **(R-I)** | **(I)** | **(R)** | **(R-I)** |
| ClustalW | 4.59% | 4.49% | **-0.10%** | 97.59% | 97.70% | **0.11%** | 92.17% | 92.63% | **0.46%** |
| ClustalO | 4.47% | 4.39% | -0.08% | 97.73% | 97.82% | **0.09%** | 92.88% | 93.20% | 0.32% |
| Kalign | 7.98% | 5.57% | **-2.41%** | 97.69% | 97.77% | **0.08%** | 92.66% | 93.08% | **0.42%** |
| Mafft (FFTnsi) | 4.30% | 4.32% | **0.02%** | 97.88% | 97.86% | **-0.02%** | 93.48% | 93.40% | **-0.08%** |
| Mafft (Linsi) | 4.39% | 4.28% | -0.11% | 97.80% | 97.90% | 0.10% | 92.91% | 93.33% | 0.42% |
| Muscle | 4.35% | 4.36% | **0.01%** | 97.83% | 97.82% | **-0.01%** | 93.14% | 93.10% | **-0.04%** |
| DialignTX | 4.52% | 4.35% | **-0.17%** | 97.66% | 97.84% | **0.18%** | 92.58% | 93.20% | **0.62%** |
| GramAlign | 12.83% | 5.04% | **-7.79%** | 88.03% | 97.15% | **9.12%** | 75.70% | 91.76% | **16.06%** |
| ProbCons | 6.83% | 5.61% | **-1.22%** | 97.84% | 97.85% | 0.01% | 93.21% | 93.37% | **0.16%** |
| PicXAA | 6.56% | 5.60% | **-0.96%** | 97.85% | 97.86% | 0.01% | 93.22% | 93.33% | **0.11%** |
| R-Coffee | 6.68% | 5.51% | **-1.17%** | 97.42% | 97.84% | **0.42%** | 91.99% | 93.34% | **1.35%** |
