# Supplementary material for: ReformAlign: improved multiple sequence alignments using a profile-based meta-alignment approach
Source: BMC Bioinformatics. 2014 Aug 7;15(1):265. doi: 10.1186/1471-2105-15-265 (PMC4133627; doi:10.1186/1471-2105-15-265)
Supplement: Supplementary file 3 — Additional file 3: Table S3: DNA SMART Results. Averaged D-POS, SP and TC scores for the 264 benchmark tests of the DNA SMART dataset. For each accuracy metric three figures are provided corresponding to the average scores of the initial alignments (I), the reformed alignments (R) and their respective differences (R-I). Statistically significant differences at the .05 significance level are highlighted in bold. (DOC 42 KB) [file 12859_2014_6534_MOESM3_ESM.doc]

## Additional file 3: Table S3 – DNA SMART Results

Averaged D-POS, SP and TC scores for the 264 benchmark tests of the DNA SMART dataset. For each accuracy metric three figures are provided corresponding to the average scores of the initial alignments (I), the reformed alignments (R) and their respective differences (R-I). Statistically significant differences at the .05 significance level are highlighted in bold.

| **APSI [25%-90%] (N=264)** | | | | | | | | | |
| --- | --- | --- | --- | --- | --- | --- | --- | --- | --- |
|  | **D-POS** | | | **SP** | | | **TC** | | |
| **Aligner** | **(I)** | **(R)** | **(R-I)** | **(I)** | **(R)** | **(R-I)** | **(I)** | **(R)** | **(R-I)** |
| ClustalW | 44.31% | 36.41% | **-7.90%** | 57.82% | 66.07% | **8.25%** | 11.78% | 18.58% | **6.80%** |
| ClustalO | 32.50% | 29.14% | **-3.36%** | 70.26% | 73.84% | **3.58%** | 23.69% | 24.34% | 0.65% |
| Kalign | 34.97% | 31.08% | **-3.89%** | 67.69% | 71.73% | **4.04%** | 16.83% | 22.36% | **5.53%** |
| Mafft (FFTnsi) | 31.63% | 28.93% | **-2.70%** | 71.24% | 74.08% | **2.84%** | 26.75% | 23.97% | **-2.78%** |
| Mafft (Linsi) | 23.71% | 25.20% | **1.49%** | 79.53% | 77.97% | **-1.56%** | 33.11% | 26.72% | **-6.39%** |
| Muscle | 34.13% | 31.50% | **-2.63%** | 68.60% | 71.35% | **2.75%** | 20.73% | 21.87% | 1.14% |
| DialignTX | 42.43% | 33.65% | **-8.78%** | 59.85% | 69.08% | **9.23%** | 13.14% | 19.27% | **6.14%** |
| GramAlign | 34.53% | 32.25% | **-2.28%** | 67.98% | 70.41% | **2.43%** | 25.32% | 25.13% | -0.19% |
| ProbCons | 32.07% | 31.07% | **-1.00%** | 70.82% | 71.81% | **0.99%** | 19.54% | 20.44% | 0.90% |
| PicXAA | 30.96% | 31.30% | **0.34%** | 71.95% | 71.56% | **-0.39%** | 20.62% | 20.40% | -0.22% |
